# Risk equations for the development of worsened glucose status and type 2 diabetes mellitus in a Swedish intervention program

**DOI:** 10.1186/1471-2458-13-1014

**Published:** 2013-10-26

**Authors:** Anne Neumann, Margareta Norberg, Olaf Schoffer, Fredrik Norström, Ingegerd Johansson, Stefanie J Klug, Lars Lindholm

**Affiliations:** 1 Epidemiology and Global Health, Department of Public Health and Clinical Medicine, Umeå University, Umeå 901 85, SE, Sweden; 2 Cancer Epidemiology, University Cancer Center, University Hospital, Technische Universität Dresden, Fetscherstr. 74, Dresden 01307, Germany; 3 Department of Odontology, Umeå University, Umeå 901 87, SE, Sweden

**Keywords:** Diabetes mellitus, type 2, Pre-diabetic state, Prevention & control, Risk factors, Glucose, Sweden, Logistic models, Factor analysis, statistical, Early intervention, Life style

## Abstract

**Background:**

Several studies investigated transitions and risk factors from impaired glucose tolerance (IGT) to type 2 diabetes mellitus (T2D). However, there is a lack of information on the probabilities to transit from normal glucose tolerance (NGT) to different pre-diabetic states and from these states to T2D. The objective of our study is to estimate these risk equations and to quantify the influence of single or combined risk factors on these transition probabilities.

**Methods:**

Individuals who participated in the VIP program twice, having the first examination at ages 30, 40 or 50 years of age between 1990 and 1999 and the second examination 10 years later were included in the analysis. Participants were grouped into five groups: NGT, impaired fasting glucose (IFG), IGT, IFG&IGT or T2D. Fourteen potential risk factors for the development of a worse glucose state (pre-diabetes or T2D) were investigated: sex, age, education, perceived health, triglyceride, blood pressure, BMI, smoking, physical activity, snus, alcohol, nutrition and family history. Analysis was conducted in two steps. Firstly, factor analysis was used to find candidate variables; and secondly, logistic regression was employed to quantify the influence of the candidate variables. Bootstrap estimations validated the models.

**Results:**

In total, 29 937 individuals were included in the analysis. Alcohol and perceived health were excluded due to the results of the factor analysis and the logistic regression respectively. Six risk equations indicating different impacts of different risk factors on the transition to a worse glucose state were estimated and validated. The impact of each risk factor depended on the starting or ending pre-diabetes state. High levels of triglyceride, hypertension and high BMI were the strongest risk factors to transit to a worsened glucose state.

**Conclusions:**

The equations could be used to identify individuals with increased risk to develop any of the three pre-diabetic states or T2D and to adapt prevention strategies.

## Background

Type 2 diabetes mellitus (T2D) is a severe disease with considerable impact on people’s wellbeing and standard of living. Worldwide prevalence of T2D is high and expected to further increase in the coming years [1]. The age-standardized incidence rate of T2D in a Swedish community between 1971 and 2001 was 3.03 cases per 100 000 [2]. The age-standardized prevalence of T2D was 2.56% for women in 1971 and 4.07% in 2001 and 2.17% for men in 1971 and 3.93% in 2001 [2]. The annual mean cost of care for a patient with T2D in Sweden was estimated to 3 602 EUR with inpatient care consuming the most resources [3]. Risk factors associated with the development of T2D are, among others, obesity, low level of physical activity or low intake of fruits and vegetables [4-6]. Fortunately, T2D is preventable. Several studies have shown that the development of T2D can be prevented or delayed by lifestyle modification [4,5,7]. Lifestyle intervention to prevent T2D is at least as effective as pharmacological treatment [6]. In a meta-analysis, studies estimated a pooled effect for all forms of lifestyle interventions with a hazard ratio of 0.51 (95% confidence interval 0.44 to 0.60), indicating a relative risk reduction of 49% for the development of T2D [6].

The natural history of T2D describes the process of the development from normal glucose tolerance (NGT) via so-called pre-diabetic states, which are characterized by higher insulin resistance and/or reduced insulin secretion, to T2D. The pre-diabetic or worsened glucose states are impaired fasting glucose (IFG), impaired glucose tolerance (IGT) and a combination of both (IFG&IGT). Subjects in any of the three states have moderate to severe insulin resistance and impaired insulin secretion, each state having distinct pathophysiologic etiologies. For a description of the states see DeFronzo and colleagues [8]. Assuming relatively short intervals, for example one year, the direct development of T2D from NGT is not likely. At some point of time, the individual will develop IGT, IFG or a combination of both before a possible transition to T2D. It is therefore intriguing and necessary to specifically look at pre-diabetic states and factors that influence their development. Pre-diabetes is an increasingly common condition [9]. It has been reported that subjects with IFG differ from those with IGT or with a combination of both. We need studies that estimate the specific impact of glycemic states on the development of T2D and that determined which factors are driving forces for this development.

Several high-quality studies exist on the transitions and its risk factors from IGT to T2D [7,10]. However, no study has yet investigated the probability of moving from NGT to pre-diabetic states and from these states to T2D. In a previous investigation, we found no study that included probabilities of moving among all the necessary states needed in a diabetes prevention model, such as NGT, IGT and/or IFG and which was based on one population [11].

The Västerbotten Intervention Program (VIP) was initiated in 1985 with the aim to reduce morbidity and mortality from cardiovascular disease and diabetes [12]. Within this program, people at ages 40, 50 and 60 living in the Swedish county of Västerbotten were invited to a health assessment and health counseling conducted by their primary care provider [12]. Thirty-year olds were also included until 1996. Every tenth year, people living in the included area were invited again and the same measurements were taken. Part of this screening was an oral glucose tolerance test, which is the gold standard for the diagnosis of T2D as well as of IGT&IFG. This test was conducted according to standards of the World Health Organization with a 75 g oral glucose load. Measurements on height, weight, blood pressure, plasma lipids and an oral glucose tolerance test were performed, and each VIP participant was asked to complete a set of questionnaires, including questions about physical activity, tobacco use and dietary habits. The VIP was described in more detail elsewhere [12].

The objective of this study was to calculate risk equations that predict 10-year transition probabilities from NGT to pre-diabetic states and from pre-diabetic states to T2D taking major risk factors into consideration.

## Methods

Individuals who participated in the VIP program twice, having the first examination at ages 30, 40 or 50 years of age between 1990 and 1999 and the second examination 10 years later were included in the analysis. Data from the regional diabetes registry DiabNorth [13] were linked to the VIP dataset and information was compared. Among patients with diabetes, 74% consented to be included in the DiabNorth register. Subjects with a diagnosis of type 1 diabetes mellitus were excluded. If the DiabNorth indicated that a person had IGT or T2D maximal two years before or after the VIP examination, the information from the DiabNorth registry replaced the glucose status of the VIP. Otherwise additional DiabNorth information was ignored. Participants were grouped into NGT, IFG, IGT, IFG&IGT or T2D by WHO classification (1999) according to the results of the oral glucose tolerance test [14].

Fourteen potential risk factors for the development of a worse glucose state (pre-diabetes or T2D) were investigated. Table 1 describes all potential risk factors considered in the study.

**Table 1:** Description of risk factors under investigation, Västerbotten Intervention Program

**Variable**	**Values**	**Description**
Sex	Male / female	
Age		Years of age, each decade includes one year older and younger, e.g. 30 = 29–31 years of age etc.
Education	High / middle / low	University OR education of >12 years in school / 10–12 years of education in school / compulsory school OR < 10 years of education in school
Marital status	Married OR living with spouse / single	Single = not married OR widowed OR divorced
Perceived health	Good / bad	Questionnaire of well-being, original score: very good, pretty good, somewhat good, pretty bad, bad; first two were merge to “good” and latter three to “bad”
Triglyceride	Normal / high	Triglyceride levels: ≤ 1.69 / ≥ 1.7 mmol/l
Blood pressure	Normal / high	Systolic blood pressure < 140 mmHg and diastolic blood pressure < 90 mmHg AND no self-reported anti-hypertensive drug / self-reported anti-hypertensive drug OR systolic blood pressure ≥ 140 mmHg OR diastolic blood pressure ≥ 90 mmHg
Body mass index (BMI)	Underweight & normal / overweight / obesity	calculation: (weight in kg) / (height in m)^2^: ≤ 24.9 / 25.0 – 29.9 / ≥ 30.0
Smoking status	Never / formerly / present	
Physical activity	Physically active / moderately active / sedentary	Physically active = exercise at least 2–3 times/week or walk and/or cycle more than 3 times/week during leisure time or walk or cycle to work more than 5 km per way
		Moderately active = do exercise now and then but not regularly or cycle and/or walk during their leisure time at least 2–3 times per week or cycle and/or walk to work 2–5 km each way
		Sedentary = never exercise or walk and/or cycle during their leisure time less than 2–3 times per week or take bus or car to work or cycle and/or walk to work less than 2 km per way.
Snus	No current use / ≤ 4 cans per week / > 4 cans per week	Snus is an oral non-smoking tobacco that is commonly used in Sweden. It is put into the mouth, usually underneath the upper lip. The biological effect of snus use is different from smoking [15].
Alcohol abuse	Normal / risk of harmful alcohol consumption	Test for harmful alcohol consumption (CAGE questionnaire: 0–1 / 2–4)
5 a day	At least 5 a day / less than 5 a day	The average consumption of the following fruits and vegetables was summed (based on Food Frequency Questionnaire): berries (fresh or frozen), apples / pears / peaches / oranges / grape, bananas, carrots, tomatoes / cucumbers, salad / spinach / broccoli;
		At least 5 a day = at least five portions of the above fruits and/or vegetables per day
		Less than 5 a day = less than five portions of the above fruits and/or vegetables per day
Family history	No parents and or siblings with T2D / parents or siblings with T2D	

The analysis was conducted in two steps. Firstly, factor analysis was used to find candidate variables; and secondly, logistic regression was employed to quantify the influence of the candidate variables.

As all risk factors have a high potential to interact, factor analysis was used to exclude multicollinear variables. Factor analysis is a statistical method that describes variability among observed, correlated variables in terms of a potentially lower number of unobserved variables called factors [16]. It describes the relation between variables. Factor loadings reveal the extent to which each of the variables contributes to the meaning of each of the factors. Uniqueness is the variance that is “unique” to the variable and not shared with other variables. Variables in our analysis were kept if they fulfilled any of the following “keep-conditions” of having either the highest factor load for one factor, low factor loadings (below ±0.55) [17] or uniqueness above 0.5.

Logistic regression (binary) was used to derive transition probabilities for movements between each of the two states. Stepwise logistic regression analyses, using backwards elimination, with a significance level of 0.2 were conducted for all possible transition probabilities here [18]. In backwards elimination, the method first includes all variables and step-by-step eliminates variables until no omitted variable would have contributed significantly to the model. Thus, the p-values of individual parameters are compared with the “stay-level”, which was 0.2. The higher significance level of 0.2 for backwards elimination prevents the model to exclude too many variables.

The results are given as odds ratios (OR) with their 95% confidence intervals (95% CI). Coefficients are employed for risk equations (see Additional file 1). Risk equations describe the relationship between the possible risk factors stating the likelihood of moving from one state to another. The relationship can be expressed by ORs or coefficients. ORs above 1 or coefficients above zero state an increased risk for an increase of the variable.

To validate the results, the bootstrap technique was used [19]. We drew, with replacement, as many individuals as the sample size from our data. The 95% CIs of the coefficients based on 1 000 repetitions was estimated by the percentile method. It was tested whether zero lied without the 95% CIs.

The software program STATA/SE 11.0 (StataCorp LP, College Station, TX) was used for analyses. SAS 9.22 (SAS Institute Inc., Cary, NC) was used for the visualization of ORs and CIs. Ethical approval for this study was received from the Regional Ethics Board Dnr 08-131 M at Umeå University, Sweden. All subjects gave informed consent to future research before their VIP-examination.

## Results

### Population

In total, 29 937 individuals were included in the analysis. Table 2 shows the age and sex distribution of the study population. Most participants were 40 or 50 years of age at their first examination. About half of the participants (53%) were women.

**Table 2:** Description of Västerbotten Intervention Program population at first examination (n = 29 937)

**Variable**	**Number**	**%**
**Sex**		
Male	13 968	46.7
Female	15 969	53.3
Missing	0	0
**Age**		
30 years	4 917	16.4
40 years	12 218	40.8
50 years	12 802	42.8
Missing	0	0
**Education**		
High	7 386	24.7
Middle	15 854	53.0
Low	6 353	21.2
Missing	344	1.1
**Marital status**		
Married/living with spouse	24 794	82.8
Single	4 786	16.0
Missing	357	1.2
**Perceived health**		
Good	22 727	75.9
Bad	6 646	22.2
Missing	564	1.9
**Triglyceride**		
Normal	18 928	63.2
High	4 551	15.2
Missing	6 458	21.6
**Blood pressure**		
Normal	23 138	77.3
High	6 500	21.7
Missing	299	1.0
**Body mass index (BMI)**		
Underweight & normal	16 281	54.4
Overweight	10 692	35.7
Obesity	2 784	9.3
Missing	180	0.6
**Smoking status**		
Never	13 753	45.9
Formerly	8 721	29.1
Present	6 976	23.3
Missing	487	1.6
**Physical activity**		
Physically active	4 018	13.4
Moderately active	20 115	67.2
Sedentary	5 384	18.0
Missing	420	1.4
**Snus**		
No current use	24 927	83.3
≤ 4 cans per week	3 293	11.0
> 4 cans per week	973	3.3
missing	744	2.5
**Alcohol abuse**		
Normal	22 927	76.6
Risk of harmful alcohol consumption	1 799	6.0
Missing	5 211	17.4
**5 a day**		
At least 5 a day	2 203	7.4
Less than 5 a day	20 303	67.8
Missing	7 431	24.8
**Family history**		
No parents or siblings with T2D	24 273	81.1
Parents or siblings with T2D	4 994	16.7
Missing	670	2.2

**Figure 1:**
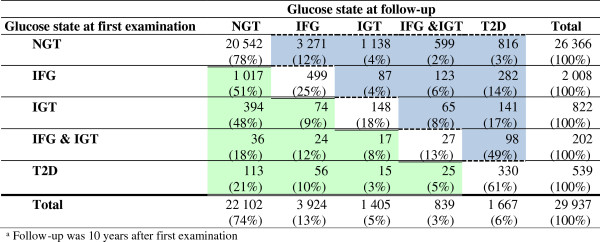
**Glucose states during first examination in the Västerbotten Intervention Program and at follow-up**^**a**^**.**

Figure 1 shows the glucose states during the first examination and during the follow-up examination in the VIP, which was 10 years later. Row percentages indicate the development of glucose states in individuals after ten years. The blue cells (dashed frame) in Figure 1 are always larger than the equivalent green complementary cells (double solid frame). This indicates that in each situation more cases move forward in the natural history of the development of T2D than backwards. We found that 12%, 4% and 2% of those individuals with NGT at first examination had moved to IFG, IGT and IFG&IGT, respectively. Further, 14%, 17% and 49% moved to T2D starting from IFG, IGT or IFG&IGT, respectively. Most individuals, however, remained in the glucose state of their first examination (NGT: 78%, T2D: 61%).

### Test to prevent multicollinearity

Factor analysis determined that the variable “risk for harmful alcohol consumption” needs to be excluded from the model as it did not fulfill any of the necessary “keep-conditions” (Table 3). Factor 1 was determined by “sex” (factor loading (fl): -0.75) and “snus” (fl: 0.67). “Snus” had uniqueness above 0.5. Factor 2 contained the variables “age” (fl: 0.70) and “blood pressure” (fl: 0.67). “Blood pressure” had uniqueness above 0.5. The variable “smoking” (fl: 0.79) represented Factor 3. Factor 4 was described by “perceived health” (fl: 0.74). Factor 5 did not entail a variable with a high factor loading. “Education”, “marital status”, “triglycerides”, “BMI”, “physical activity”, “consumption of at least 5 portions of fruits or vegetables a day” and “family history” revealed fair to poor factor loadings (below ±0.55). All seven variables were kept in the model due to their relatively low chance for multicollinearity. Only the highest factor loading of each variable is displayed (Table 3).

**Table 3:** **Results from factor analysis showing highest factor loading and uniqueness per variable**^**a**^

**Variable**	**Factor 1**	**Factor 2**	**Factor 3**	**Factor 4**	**Factor 5**	**Uniqueness**
**Sex**^**b**^	**-0.75**					0.35
**Age**^**b**^		**0.70**				0.38
**Education**^**cd**^			0.49			0.56
**Marital Status**^**cd**^				0.38		0.67
**Perceived Health**^**b**^				**0.74**		0.43
**Triglyceride**^**cd**^		0.37				0.59
**Blood Pressure**^**d**^		0.67				0.52
**BMI**^**c**^		0.48				0.46
**Smoking**^**b**^			**0.79**			0.37
**Physical Activity**^**cd**^					0.53	0.56
**Snus**^**d**^	0.67					0.52
**Risk of Harmful Alcohol Consumption**^**e**^	0.57					0.46
**5 a day**^**bcdf**^					**0.54**	0.56
**Family history**^**cd**^				0.25		0.82

^a^Numbers in bold signify the highest factor loading of each factor.

^b^Highest factor loading of factor.

^c^Variable with factor loadings below ±0.55. Variable will be kept in the model.

^d^Variable with uniqueness above 0.5. Variable will be kept in the model.

^e^Variable removed.

^f^5 a day = consumption of at least five portions of fruits and vegetables a day.

### Establishment of risk equations

**Figure 2:**
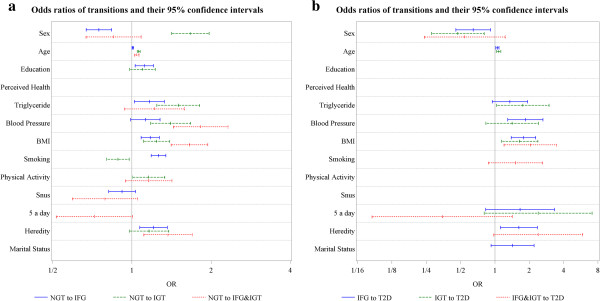
a: Odds ratios (OR) of progression from NGT to IFG, IGT and IFG&IGT and their 95% confidence intervals by risk factors in a logarithmic scale; b: Odds ratios (OR) of progression from IFG, IGT and IFG&IGT to T2D and their 95% confidence intervals by risk factors in a logarithmic scale.

The results of stepwise logistic regressions for the individual contribution of each risk factor with backwards elimination are shown in Table 4. See Additional file 1 for risk equations with coefficients. The reference (OR = 1) for each variable is specified. The backwards regression analyses removed those risk factors from the 13 potential factors in every model equation that did not fulfill the 0.2 significance level. The variable perceived health was excluded in every of the six regression models through backwards elimination and is consequently not included in further analyses. Odds ratios and 95% CIs for each risk factor and each transition are shown in a logarithmic scale in Figure 2a and b.

**Table 4:** Odds ratios (95% confidence interval) of risk to progression to another state by risk factors, stepwise logistic regression (backwards elimination, significance level = 0.2)

**Risk factors**												
**From state A to state B**	**Sex**	**Age**	**Education**	**Triglyceride**	**Blood pressure**	**BMI**	**Smoking**	**Physical activity**	**Snus**	**5 a day**	**Marital status**	**Family history**
***Reference (non-exposure)***	***Male***	***30 years***	***High***	***Normal***	***Normal***	***Under-weight/normal***	***Never***	***Physically active***	***No current use***	***At least 5 a day***	***Married or living with spouse***	***No Parents and/or siblings with T2D***
**NGT to IFG**	0.75^a^ (0.67-0.84)	1.01^a^ (>1.00-1.02)	1.11^a^ (1.03-1.21)	1.17^a^ (1.02-1.33)	1.12 (0.99-1.28)	1.17^a^ (1.03-1.27)	1.26^a^ (1.18-1.35)	n.a.	0.92 (0.82-1.03)	n.a.	n.a.	1.21^a^ (1.07-1.36)
n = 15 473												
**NGT to IGT**	1.67^a^ (1.41-1.97)	1.06^a^ (1.05-1.07)	1.10 (0.98-1.23)	1.50^a^ (1.24-1.81)	1.40^a^ (1.18-1.67)	1.24^a^ (1.11-1.39)	0.89^a^ (0.80-0.98)	1.16^a^ (>1.00-1.33)	n.a.	n.a.	n.a.	1.16 (0.98-1.38)
n = 15 473												
**NGT to IFG&IGT**	0.85 (0.67-1.08)	1.04^a^ (1.03-1.06)	n.a.	1.22 (0.94-1.58)	1.83^a^ (1.44-2.32)	1.66^a^ (1.41-1.94)	n.a.	1.16 (0.95-1.42)	0.79 (0.59-1.05)	0.72 (0.52- > 1.00)	n.a.	1.37^a^ (1.11-1.69)
n = 15 473												
**IFG to T2D**	0.65^a^ (0.45-0.92)	1.05^a^ (1.02-1.09)	n.a.	1.35 (0.95-1.93)	1.85^a^ (1.29-2.64)	1.78^a^ (1.39-2.27)	n.a.	n.a.	n.a.	1.58 (0.82-3.05)	1.43 (0.93-2.20)	1.66 (0.83-3.33)
n = 1 299												
**IGT to T2D**	0.47^a^ (0.28-0.80)	1.08^a^ (1.03-1.13)	n.a.	1.76^a^ (1.04-2.98)	1.42 (0.83-2.40)	1.65^a^ (1.14-2.38)	n.a.	n.a.	n.a.	2.39 (0.81-7.10)	n.a.	n.a.
n = 460												
**IFG&IGT to T2D**	0.54 (0.24-1.23)	n.a.	n.a.	n.a.	n.a.	2.04^a^ (1.20-3.45)	1.52 (0.88-2.63)	n.a.	n.a.	0.35 (0.08-1.43)	n.a.	2.40 (0.98-5.87)
n = 120												

^a^ Statistically significant (5% significance level, Wald-test).

*Sex* (1 = male, 2 = female), *Education* (1 = high, 2 = middle, 3 = low), *Perceived health* (1 = very good, pretty good, 2 = somewhat good, pretty bad, bad), *Triglyceride* (0 = normal (TG ≤ 1.69 mmol/l), 1 = high (TG ≥ 1.7 mmol/l)), *Blood pressure* (0 = normal (systolic blood pressure < 140 mmHg and diastolic blood pressure < 90 mmHg and no self-reported anti-hypertensive drug), 1 = self-reported anti-hypertensive drug OR systolic blood pressure ≥ 140 mmHg OR diastolic blood pressure ≥ 90 mmHg), *BMI* (1 = underweight/normal (≤ 25), 2 = overweight (25–29.9), 3 = obese/severely obese (≥ 30)), *Smoking* (1 = never, 2 = formerly, 3 = present), *Physical activity* (0 = physically active (exercise at least 2–3 times/week or walk and/or cycle more than 3 times/week during leisure time or walk or cycle to work more than 5 km per way), 1 = moderately active (do exercise now and then but not regularly or cycle and/or walk during their leisure time at least 2–3 times per week or cycle and/or walk to work 2–5 km each way), 2 = sedentary (never exercise or walk and/or cycle during their leisure time less than 2–3 times per week or take bus or car to work or cycle and/or walk to work less than 2 km per way), *Snus* (1 = no current use, 2 = maximal 4 cans/week, 3 = more than 4 cans/week), *Alcohol* (0 = no risk of harmful alcohol consumption, 1 = risk of harmful alcohol consumption), *5 a day* (1 = at least five portions of fruits or vegetables per day, at 2 = less than five portions of fruits or vegetables per day), *Marital status* (1 = married or living with spouse, 2 = single or widowed or divorced), *Family history* (1 = no parents and/or siblings with T2D, 2 = parents and siblings with T2D).

n.a. = variable was removed from logistic regression by backward elimination.

The low number of individuals who had IFG&IGT at baseline examination lowered the chance of statistical significance. In fact, only BMI was statistically significant in the transition from IFG&IGT to T2D.

With the tools created here, it is possible to calculate different scenarios adapting a specific risk profile. For example, the change in risk could be estimated for a woman with increased consumption of fruits and vegetables, a change from high levels of triglyceride to normal levels, a change from hypertension to normal blood pressure and a reduction of weight (Additional file 1). We can estimate how the risk to develop any of the worsened glucose states or T2D changes by altering any of the risk factors in the model.

## Discussion

This study developed risk equations from healthy (NGT) to pre-diabetic states and from pre-diabetes states to T2D using data of a Swedish population. In total, six risk equations were developed and validated. The equations can be used to identify individuals with increased risk to develop any of the three pre-diabetic states or T2D. In addition, the equations are useful for adapting prevention strategies to specific risk profiles. Risk models are widely used in clinical and public health practice [20]. The six risk equations used in this study describe the risk of developing a pre-diabetic state from being healthy as well as developing T2D depending on modifiable and non-modifiable risk factors. The risk equations allow adjustment to a specific risk profile and thus give more precise risk estimates than general risk models.

We found that 49% of those with IFG&IGT at baseline developed T2D in comparison to 3%, 14% and 17% for those with NGT, IFG and IGT (Figure 1). Other prospective studies have also found that a combination of IFG and IGT increases the risk of developing T2D compared to subjects having either of the glycemic abnormalities [9,21-23]. For example, de Vegt and colleagues estimated that the ORs for T2D were 10.1, 10.9 and 39.5 for those having IFG, IGT and IFG&IGT, respectively [21].

From the results of the logistic regression, it seems that the variables snus and sex as well as the variables education and smoking might show multicollinearity. However, we have examined the influence of each pair of risk factors and could not find that this first impression was true. All four potential risk factors were hence kept in the logistic regression estimations. We also compared whether OR coefficients and their equivalent bootstrap results in all logistic regression models were significantly different from 1. Besides the variables age and triglyceride in the transition from NGT to IFG as well as the variable “five a day” in the transitions from NGT to IFG&IGT and from IGT to T2D, all other variables could be validated with the bootstrap estimations.

### Risk factors

In our study, sex had different influence on the progression to a pre-diabetic state. Whereas men have a higher risk to develop IFG, women have higher risk to develop IGT. As expected, the progression from NGT to IGT and/or IFG exhibits striking sex differences [24-26]. In most populations, IFG is substantially more common amongst men and IGT is slightly more common amongst women [26,27]. In a study from Turkey, however, IFG and IGT were more common in women than in men [27]. Meigs and colleagues reported that men in comparison to women had a higher risk to progress from NGT to IFG and/or IGT in the United States [28].

As expected, increasing age increases the likelihood to develop a worse diabetic state. Even though age is a non-modifiable risk factor, it needs to be included in all risk equations.

Our data suggest that lower education increases the risk to develop IFG from NGT, even tough the confidence interval of the odds ratio is quite close to one (non-significance). Nonetheless, education might be an important player in prevention of a pre-diabetic state.

Self-rated health was excluded from all six risk equations indicating that it did not add to the models. However, T2D is known to be related to low self-rated health [29,30]. However, our results might be due to a small sample size for the equation to or from IFG&IGT or due to difficulties in measuring self-rated health.

As expected, high triglyceride levels, high blood pressure and high BMI are the strongest factors for a progression to a worse diabetic state. All are well-known risk factors for the development of T2D. In a study by Jauch-Chara and colleagues, low body weight was associated with increased risk to develop IGT from NGT [31]. Underweight could not be examined separately in our study but was combined with normal weight due to the low number of cases in the underweight category. The influence of low body weight could thus not be estimated with our data. Our risk models assume a linear relationship looking only at increased body weight. In another study, BMI and waist circumference were higher in subjects with abnormalities of glucose metabolism compared to NGT [32]. A study from the United States also found that higher BMI increased the rate to progress from NGT to IFG and/or IGT [28].

The odds ratio of smoking was above 1 for the development of NGT to IFG and below 1 for the development of NGT to IGT. In a study with American Indians, participants with pre-diabetes reported significantly less smoking than participants with NGT and were significantly more likely to be past smokers [9]. However, in our study smokers and past-smokers were relatively evenly distributed at first examination. Our population included 24%, 29%, 19%, 21% and 29% smokers and 29%, 31%, 30%, 37% and 29% past-smokers among NGT, IFG, IGT, IFG&IGT and T2D respectively. In addition, smoking is related to lower BMI. Smoking prevalence has decreased and prevalence of high BMI has increased over time in this population. Individuals must have, therefore, quit smoking between baseline and follow-up. Smoking cessation might lead to an increased in weight and BMI [33]. Possibly, BMI could also be the explanatory factor here.

Lower level of physical activity (vs. higher level) slightly increased the risk to develop IGT from NGT. This is consistent with the literature [4,5].

The odds ratios of snus, “five a day” and marital status were all not significant. We aimed to describe diet with one simple variable in our model. However, the question what is healthy diet is difficult to answer. The purpose of the variable we created was that it needed to be simple and easy to understand. We decided to use the consumption of five portions of fruits and vegetables a day as a proxy of healthy diet, knowing that this is a simplification of reality. Diet is far more complex.

Marital status did not have any significant impact on the development of a worsened glucose status. It was only included in the model IFG to T2D but could not reach statistical significance.

In our population, individuals with a family history of diabetes developed a worsened glucose status more likely than those without a family history of diabetes. This factor was only excluded in the development from IGT to T2D. An evaluation of the Stockholm Diabetes Prevention Program, however, demonstrated that prevalence of IFG, similarly to the prevalence of IGT, IFG and IGT combined and T2D, was nearly twofold higher in those who had a family history of T2D compared to those without family history of T2D [32]. It needs to be kept in mind that knowledge about family medical status and age of respondent might have been important influences on whether the study participant reported a family history of T2D. For example, it has been shown in our VIP population that knowledge about family history is rather low, in particular among younger men [34]. In addition, parents of young study participants might not have been diagnosed with T2D yet [34].

In consequence, the influence of specific risk factors on the transition to worse states towards the development of T2D is diverse. Different risk factors have different impacts on the development of IFG, IGT, IFG&IGT and T2D.

### Use of results in practice

Once glucose status has been estimated, information used to perform risk equations are relatively easy to obtain, for example age, smoking status or measurement of BMI. For the classification of the glucose status, an oral glucose tolerance test is needed. This need of a test is a challenge, because in a practical setting the individual or their physician rarely knows the patient’s glucose status. The advantage of our risk equations over similar risk tools is that four different glucose states can be represented, establishing six unique risk equations [33]. Other risk equations focused on the development of T2D only [35-37].

As Noble and colleagues pointed out, caution is needed when extrapolating risk models to a different population [20]. The models, therefore, best describe the Swedish population. Risk equations should be evaluated to determine whether they are also valid in other populations and in other prospective cohorts.

### Combined risk

Comparing our results without looking at risk factors leads us back to Figure 1. Among individuals with IFG or IGT at baseline, 14% and 17%, respectively, developed T2D 10 years later. For those with a combination of IFG and IGT the risk was much higher. Almost half (49%) of our population with IFG&IGT at baseline developed T2D within 10 years. Our overall one-year risks for T2D (estimated based on 10-year changes within our cohort [38]) were 1.5%, 1.8% and 6.5% for IFG, IGT and IFG&IGT respectively.

In other studies, the annual progression rates to T2D were 1-5% for individuals with IFG and 3-11% for those with IGT [21,23,39,40]. The Hoorn study estimated that the annual rate of developing T2D from IFG alone was 5.5%, and from IFG together with IGT 10.8% [21]. The Paris Prospective Study reported a much lower annual rate, with 1% for individuals with IFG and 6% for individuals with IFG and IGT [22]. An Italian study estimated an annual rate of 0.8% to develop diabetes from IFG alone and an annual rate of 3.9% to develop diabetes from a combination of IFG and IGT [23]. A study from Iran showed that patients with first-degree relatives with T2D have a risk of 8.6% to progress to IFG and a risk of 3.7% to progress to IGT [41]. Our results are comparable with the Paris Prospective Study and the Italian study. Nonetheless, these different risks point out that the risk profile of different populations is quite diverse. We examined a Swedish population from a population-based perspective, meaning that we did not aim at any high-risk profile population but intended to investigate the general public. This might be one reason why progression rates in our study are rather low in comparison with other studies. The highest risk to develop T2D was presented by combined IFG and IGT [42]. Further, VIP participants have participated in interventions that aimed at the reduction of T2D and cardiovascular disease. They had experienced motivational counseling regarding life style modification. This might have contributed to comparable low rates of progression.

### Limitations

Even though a high number of individuals could be enclosed in this modeling study, some risk equations only included a small number of study participants, such as in the risk equation from IFG&IGT to T2D. This definitely hampered the ability to depict variables as potential risk factors.

Due to the design of the VIP, the number of people with T2D is likely to be underestimated. We included only panel data with information at baseline and 10 years follow-up. If a person was diagnosed with T2D at first or between first and second examination, he or she was less likely to participate in the VIP or in the second VIP examination. As a consequence, more people can be expected to be in T2D at baseline and at follow-up. However, any change in status between first and second examination of those individuals who participated twice were caught by the DiabNorth register. Also, our risk equations only consider worsening of the glucose state, not the reverse direction towards a less deteriorated glucose state.

In addition, we could only calculate risk over a 10-year period. Many events can happen during such a long time period. For example, individuals who would have developed any pre-diabetic state after some years could have progressed to T2D within the 10 years or someone has been in a pre-diabetic state and returned to NGT after 10 years. Also, a substantial number of individuals with IFG or IGT revert back to NGT [43]. These changes between the states within the 10-year time frame could not be traced in our study, unless the participant was included in the diabetes registry. The progression to another state, nonetheless, takes many years. Meigs and colleagues suggest that subjects with IFG and IGT are already close to transitioning to T2D and underline that T2D develops slowly over many years, transitioning through a prolonged state of impaired glycemia [28]. Also, individuals who were registered in the DiabNorth register would have been traced and re-sorted according to information in the DiabNorth.

## Conclusion

Our research has established and validated risk equations describing the development from a healthy individual to a pre-diabetic state, and from a pre-diabetic state to T2D. It clearly shows that, on the one hand, the risk to develop a worsened glucose state depends on the glucose state at baseline. On the other hand, the risk also depends on several well-established risk factors whose influence differs depending on the glucose state at baseline.

The equations are based on a population from the north of Sweden and are expected to work well in other parts of Europe, too. However, the models need to be confirmed for other populations. The risk equations help to describe an individual risk. They quantify the influence of modifiable and non-modifiable risk factors. This will help to investigate the influence of single and combined risk factors on the development of T2D through its pre-diabetic states.

As the number are small in some risk equations, such as for IFG&IGT to T2D, further studies on the influence of specific glucose statuses on the development of worsened glucose status are needed to finally advance prevention and treatment in this area.

## Abbreviations

IFG: Impaired fasting glucose; IGT: Impaired glucose tolerance; NGT: Normal glucose tolerance; T2D: Type 2 diabetes mellitus; VIP: Västerbotten Intervention Program.

## Competing interests

The authors declare that they have no competing interests.

## Authors’ contributions

AN drafted the design of the study, did statistical analyses, drafted and revised the manuscript. MN advised in acquisition and handling of data and in diabetes science, and revised the manuscript critically. OS discussed methodological aspects, gave statistical advice and programed SAS graphs. FN gave statistical advice and revised the manuscript critically. IJ advised on nutritional aspects and on how to use nutritional information in the risk equations and revised the manuscript critically. SJK discussed the project and revised the manuscript critically. LL advised on the design of the study and on data handling and revised the manuscript critically. All authors read and approved the final manuscript.

## Pre-publication history

The pre-publication history for this paper can be accessed here:

http://www.biomedcentral.com/1471-2458/13/1014/prepub

## Supplementary Material

Additional file 1 Risk equations for moving to/from pre-diabetic states by risk factors, stepwise logistic regression (backwards elimination, logit function, significance level = 0.2). Click here for file
