# Supplementary material for: Risk equations for the development of worsened glucose status and type 2 diabetes mellitus in a Swedish intervention program
Source: BMC Public Health. 2013 Oct 26;13:1014. doi: 10.1186/1471-2458-13-1014 (PMC3871001; doi:10.1186/1471-2458-13-1014)
Supplement: Additional file 1 — Risk equations for moving to/from pre-diabetic states by risk factors, stepwise logistic regression (backwards elimination, logit function, significance level = 0.2). [file 1471-2458-13-1014-S1.docx]

Supplementary material: Risk equations for moving to/from pre-diabetic states by risk factors, stepwise logistic regression (backwards elimination, logit function, significance level = 0.2)

| **From state A to state B** | **Regression model** |
| --- | --- |
| **NGT to IFG** | Logit P(NGT to IFG) = - 3.07 - 0.29 * sex + 0.01 * age + 0.11 * education + 0.19 * heredity - 0.09 * snus + 0.15 * triglyceride + 0.12 * blood pressure + 0.16 * BMI + 0.23 * smoking |
| **NGT to IGT** | Logit P(NGT to IGT) = - 7.29 + 0.51 * sex + 0.06 * age + 0.09 * education + 0.14 * physical activity + 0.15 * heredity + 0.41 * triglyceride + 0.34 * blood pressure + 0.21 * BMI - 0.12 * smoking |
| **NGT to IFG&IGT** | Logit P(NGT to IFG&IGT) = - 6.20 – 0.16 * sex + 0.04 * age - 0.24 * snus + 0.32 * heredity + 0.15 * physical activity + 0.20 * triglyceride + 0.60 * blood pressure + 0.50 * BMI - 0.33 * five a day |
| **IFG to T2D** | Logit P(IFG to T2D) = - 6.98 - 0.44 * sex + 0.05 * age + 0.48 * heredity + 0.36 * marital status + 0.51 * five a day + 0.30 * triglyceride + 0.61 * blood pressure + 0.57 * BMI |
| **IGT to T2D** | Logit P(IGT to T2D) = - 6.54 – 0.75 * sex + 0.07 * age + 0.87 * five a day + 0.35 * BMI + 0.35 * blood pressure + 0.56 * triglyceride |
| **IFG&IGT to T2D** | Logit P(IFG&IGT to T2D) = - 0.55 - 0.61 * sex + 0.71 * BMI + 0.87 * heredity - 1.06 * five a day + 0.42 * smoking |

*Example calculation 1 (IFG to T2D)*

For example, what is the 10-year risk for a woman with IFG to develop T2D if the woman has the following characteristics: age 50 year, siblings with T2D, married, not consuming at least five portions of fruits and vegetables a day, high levels of triglyceride, hypertension and BMI of 26.5?

Logit P(IFG to T2D) = - 6.98 - 0.44 * 2 + 0.05 * 50 + 0.48 * 2 + 0.36 * 1 + 0.51 * 2 + 0.30 * 1 + 0.61 * 1 + 0.57 * 2 = - 0.97

↔ P(IFG to T2D) = 1/(1+e^+0.97^) = 0.2749

Answer: The risk for this woman to develop T2D in the coming 10 years is 27.49%.

*Example calculation 2 (IFG to T2D)*

What is the 10-year risk for a woman with IFG to develop T2D if the woman has the following characteristics: age 50 year, siblings with T2D, married, consuming at least five portions of fruits and vegetables a day, normal levels of triglyceride, normal blood pressure and BMI of 24?

Logit P(IFG to T2D) = - 6.98 - 0.44 * 2 + 0.05 * 50 + 0.48 * 2 + 0.36 * 1 + 0.51 * 1 + 0.30 * 0 + 0.61 * 0 + 0.57 * 1 = -2.96

↔ P(IFG to T2D) = 1/(1+e^+2.96^) = 0.0493

Answer: The risk for this woman to develop T2D in the coming 10 years is now only 4.93%.
